# A Rapid Review of Randomized Trials Assessing the Effects of High-Intensity Interval Training on Depressive Symptoms in People with Mental Illness

**DOI:** 10.3390/ijerph191710581

**Published:** 2022-08-25

**Authors:** Jéssica Alves Ribeiro, Felipe Barreto Schuch, Kleber F. Meneghel Vargas, Paulo T. Müller, Daniel Boullosa

**Affiliations:** 1 Program of Movement Sciences, Federal University of Mato Grosso do Sul, Campo Grande 79070-900, Brazil; 2 Department of Sports Methods and Techniques, Federal University of Santa Maria, Santa Maria 97105, Brazil; 3 Maria Aparecida Pedrossian Hospital, Federal University of Mato Grosso do Sul, Campo Grande 79070-900, Brazil

**Keywords:** high-intensity interval training, sprint interval training, depression, mood disorders, mental illness, exercise

## Abstract

Objective: to systematically search for randomized controlled trials comparing the effects of high-intensity interval training (HIIT) protocols vs. control without exercise or other exercise protocols, in patients with mental disorders experiencing depressive symptoms, and to provide some guidance based on the current HIIT literature to improve further interventions. Methods: we searched for relevant studies, published by 18 August 2022 on PubMed, Embase, PsycINFO and SPORTDiscus databases, that used a HIIT protocol, involving adults (≥18 years) with a diagnosis of a mental disorder, participating in a HIIT or a control condition, and assessed for depressive symptoms. Results: Four studies accounting for 108 participants (*n* HIIT = 55; *n* comparison groups = 53) met the inclusion criteria. Three out of the four studies included found significant improvements of depressive symptoms after 12 days to 8 weeks of intervention. However, there were no differences to other forms of low-to-moderate continuous exercise in 2/3 studies. Conclusions: The limited evidence suggests the effectiveness of HIIT interventions for improving depressive symptoms in people with mental illness. However, HIIT was not superior to other exercise treatments, although a trend for its superiority may be recognized. A number of methodological issues should be considered in further interventions to better characterize and identify the most efficient HIIT modalities for the treatment of depressive symptoms in these patients.

## 1. Introduction

Physical activity (PA) and physical exercise are non-pharmacological means to improve physical and mental health in the general population and in those with mental illness [1]. Despite the well-known benefits of PA and exercise, people living with a mental illness are about 50% less likely to achieve the recommended PA levels and spend a greater amount of time in a sitting position [2]. Therefore, the identification of specific and efficient PA and exercise interventions for those with mental illness is warranted.

Among the different exercise methods used for this purpose (e.g., continuous aerobic training, resistance training), high-intensity interval training (HIIT) has recently emerged as an appealing option because it has demonstrated faster physiological and psychological benefits after a few sessions in both healthy individuals and those with mental health conditions [3,4]. Specifically, HIIT may confer some advantages for those affected by mental disorders with limited adherence to treatment options, as it is an efficient exercise modality for achieving positive outcomes with less time when compared to others forms of exercise [5].

HIIT consists of repeated bouts of cyclic exercises at intensities above the anaerobic threshold or the critical power/velocity, interspersed with active or passive recovery intervals [6]. Although there is no full consensus regarding the HIIT methods and its taxonomy [7], we could also highlight sprint interval training (SIT), another HIIT modality which consists of short (≤45 s) “all out” and non “all out” efforts [6]. Recent evidence suggests differences between different HIIT and SIT schemes regarding acute physiological and psychological responses, including metabolic fatigue and its associated affective responses during exercise [8,9,10,11]. Also, a recent review has suggested that HIIT can improve physical outcomes and reduce depressive symptoms of people with mental disorders [3]. However, the analysis of depressive symptoms in this review [3] relied on four effects, three of which were from the same trial [12,13,14]. Furthermore, this review [3] has not evaluated whether the HIIT modalities used are in agreement with current recommendations for HIIT and SIT [6,15]. This information would help to guide further research and clinical practice in those willing to implement HIIT protocols for patients with mental disorders experiencing depressive symptoms.

Thus, the aim of the current rapid review was to systematically search for randomized controlled trials comparing the effects of HIIT protocols vs. control without exercise or other exercise protocols, in patients with mental disorders experiencing depressive symptoms. Subsequently, we would verify if the HIIT protocols used agree with the current state of the art in HIIT literature to provide further guidance.

## 2. Materials and Methods

This is a rapid systematic review [16]. The protocol has not been registered, but we have followed the PRISMA statement [17].

### 2.1. Eligibility Criteria

Studies included were studies which: (1) used a HIIT protocol involving repeated bouts of any cyclic exercise at high intensity (above the anaerobic threshold or critical power/velocity), interspersed with active or passive rest intervals [6]; (2) involved adults (≥18 years) with a diagnosis of a mental disorder (e.g., anxiety disorder, bipolar disorder, depressive disorders, substance use disorder, psychotic disorders, etc.) assessed using a diagnostic tool or by a trained psychiatrist; (3) randomized participants into HIIT or a control condition; and (4) assessed depressive symptoms pre- and post- intervention. We excluded studies involving people with other comorbid physical conditions to better isolate the effect of HIIT protocols on depressive symptoms.

### 2.2. Information Sources and Search Strategy

We searched for relevant studies published by 18 August 2022 on PubMed, Embase, PsycINFO and SPORTDiscus databases. The search strategy used on PubMed was: (“high aerobic intensity training” OR “high-intensity interval training” OR “high intensity intermittent exercise” OR “intensity training” OR sprint * OR “high intensity interval training” OR “intensity exercise” OR “intermittent exercise” OR “interval training” OR “interval exercise” OR HIIT OR “circuit training” OR “high velocity” OR “fitness training” OR “intensity resistance” OR fartlek) AND (schizophrenia OR psychosis OR “psychotic disorder” OR “Schizoaffective disorder” OR “bipolar disorder” OR “major depressive disorder” OR “major depression” OR “depressive illness” OR “clinical depression” OR “mental disorder” OR “Mental illness” OR “Severe mental illness” OR anxiety OR depression OR substance abuse OR drugs). The search strategy was slightly adapted for the other databases but used the same terms. References of included studies were also revised.

### 2.3. Study Selection

One experienced reviewer (FBS) searched for relevant articles. Firstly, the duplicates were removed. Subsequently, titles and abstracts were screened. Those clearly not relevant were excluded. The full text of those that were clearly relevant, or whose relevance was unclear were read and subsequently included/excluded.

### 2.4. Data Collection Process

The same experienced reviewer (FBS) extracted data on study participants, exercise and control protocols, and outcomes. The flow chart is presented in Supplementary Figure S1.

### 2.5. Data Synthesis

A qualitative synthesis was applied. Results are presented and discussed regarding sample size, diagnosis, groups comparison, exercise loading, and main findings.

## 3. Results

A total of 3100 titles and abstracts were identified in our searches. Of these, 1194 were duplicated and 2006 titles and abstracts were screened for eligibility. After screening, four studies accounting for 108 participants (*n* HIIT = 55; *n* comparison groups = 53) met the inclusion criteria and were subsequently included for assessment (see Table 1).

The studies included evaluated the effects of HIIT on depressive symptoms in samples of people with a major depressive disorder [12], anxiety disorders [18], substance abuse [19], and a mixture of people with severe mental illnesses [20]. Of these, two compared HIIT with moderate intensity continuous exercise (MICT) [12,20], one compared HIIT with a mixture of exercise and recreational activities (i.e., placebo) [19], and another compared HIIT with low-intensity training (LIT) [18]. Three of the studies monitored HIIT intensities by means of HR responses [18,19,20], while one followed metabolic calculations [12]. Only one study [12] assessed PA (via a questionnaire) pre- to post-interventions revealing an increased PA in both experimental groups but with greater increments observed after the moderate intensity group. Meanwhile, another study assessed PA levels with accelerometry only at baseline to verify similar PA patterns between groups [20].

The majority of the studies (75%, *n* = 3/4) [12,18,19] found that HIIT interventions produced significant positive effects on depressive symptoms from pre- to post-interventions. Specifically, the study by Chapman et al. [20] was the only study that showed no statistically significant differences between pre- and post-intervention depression symptoms for either HIIT (*p* = 0.09) and MICT (*p* = 0.77). Meanwhile, the study by Plag et al. [18] showed a significant interaction with a stronger reduction in HIIT compared to LIT (*p* = 0.02). The study by Gerber et al. [12] showed a significant effect of time (*p* < 0.001) but with no differences between groups (*p* = 0.2). Finally, the study by Flemmen et al. [19] showed only a significant difference for the HIIT group (*p* < 0.05) but without between-group differences.

## 4. Discussion

The literature evaluating the effects of HIIT in people with mental disorders is still in its infancy. Currently, we have located only four trials including a total of 55 participants in the HIIT interventions. Overall, the findings are supportive of the positive effects of HIIT reducing depressive symptoms in people with severe mental illness over different time frames (from 12 days to 8 weeks) [12,18,19]. Paradoxically, the longer intervention of 12 weeks did not result in significant (*p* = 0.09) changes in depressive symptoms, although this may be due to the reduced sample size (*n* = 4) [20]. In addition, early evidence suggests that HIIT is not better than low-intensity or moderate exercise, although a trend for its superiority may be recognized when considering within and between-groups differences.

Previous studies have suggested that HIIT may be included as an easy and effective exercise modality for people with mental disorders [3]. Our findings are in line with the previous studies demonstrating that HIIT may help alleviate depressive symptoms [4]. Although the evidence is limited, the extant literature supports the notion that HIIT may alleviate depressive symptoms in those with a mental illness. The findings are of clinical importance as HIIT includes shorter sessions with similar or even faster effects on cardiometabolic outcomes when compared to MICT [15]. Meanwhile, HIIT has been alleged as being associated with negative affective responses [21]. However, exercise preferences and enjoyment vary in the general and clinical populations and some forms of HIIT may be more enjoyable and preferred over MICT for some people [22,23]. Furthermore, a recent, systematic review reported better affective responses during SIT with short sprints (5–6 s) (sSIT) when compared to other training protocols of longer sprints (15–30 s). Therefore, future research in this area should evaluate this effectiveness, along with the enjoyment and adherence of patients, to better understand the true efficiency of this training modality and its variants. It is especially important to deal with the problems of adherence to exercise of patients with mental disorders.

When analyzing the methods described in the studies found, there are a number of issues which deserve to be addressed in future studies. First, the description of the training loads used are poor and insufficient to guarantee their replication. Therefore, future studies should describe both the external (e.g., Watts) and internal loads (e.g., HR) used to better understand the fidelity to the HIIT modality [24]. This is especially important when using HIIT modalities at supramaximal intensities in which maximal and near-maximum external loads can be achieved with moderate cardiovascular responses [11]. In this regard, the identification of the anaerobic threshold or the critical power/velocity, the velocity/power associated to VO_2_max, and peak power/speed would be necessary for a more precise HIIT prescription [6,7]. Second, the use of different exercise modes at the same time [19] and the absence of control of incidental physical activity changes in most studies [18,19,20] are relevant issues which should be avoided in future interventions. This control relies on the importance of identifying the relative effect of each physical activity component on the desired outcomes [25,26,27], and the need for increasing the incidental PA to take it out of the experimental setting [12]. Third, none of the studies found evaluated the effect of HIIT on different neurotransmitters or inflammatory mediators, of which it has been previously suggested that they are related to the physiopathology of depression [28,29,30]. Therefore, future studies evaluating these molecular responses should be conducted to identify the mechanisms by which different HIIT schemes improve depressive symptoms.

The present review has some limitations. First, we included a small number of studies, and the studies were too heterogeneous in their methods. For example, only two studies used the same comparison group (MICT), but the comparison group can heavily influence the magnitude of the effect sizes [31]. According to a previous systematic review, low-intensity exercises have non-significant effects, while moderate and intense exercises have great effects on depressive symptoms in people with depression [32]. Second, the trials used small sample sizes and were not designed to test the non-inferiority of HIIT versus MICT, therefore, the evidence showing lack of differences between both is weak and may change with further studies. Third, we were not able to evaluate the risk of publication bias. Thus, it would be important that researchers report both negative and positive outcomes of the different forms of HIIT to better understand the true effectiveness of this promising therapeutic tool.

From a practical point of view, the limited number of studies and their heterogeneity only allow for the suggestion that brief, high-intensity cyclic protocols (e.g., 20–30 min of treadmill or bike), with bouts from 30 s to 4 min, three times per week, during a few weeks (>4 weeks) may be sufficient to significantly improve depressive symptoms in patients with mental illnesses. This is a relevant information for the treatment of mental disorders as these exercise protocols are feasible and time-efficient when applied by an exercise professional.

## 5. Conclusions

In conclusion, the current limited evidence supports the efficacy of HIIT to reduce depressive symptoms in patients with mental disorders. However, HIIT is not more effective than other exercise interventions, although a trend for its superiority may be recognized. More studies are warranted to better understand the physical, psychological, and physiological impact of different HIIT modalities on these patients, to identify the best protocols to efficiently reduce their symptoms of depression. The quality of future studies will improve when using greater sample sizes while better characterizing the exercise protocols used.

## Figures and Tables

**Table 1:** Studies’ characteristics and outcomes.

	Sample	Depression Scale	Comparison	*n* HIIT/Comparison	Sex HIIT/Comparison (% Women)	Age HIIT/Comparison (Years)	Frequency	Trial Duration (Weeks)	Session Time (Minutes)		Bout/Rest Time (Seconds)	Intensity (Bout)	Intensity (Rest)	Outcome
Chapman et al., 2017 [20]	Mental illness	Depression Anxiety Stress Scale (DASS21)	MIT	4/5	58/4	37.4/38.6	3	12	20	bike	240/60	85–95% peak HR	60–70% peak HR	No significant improvements. No between-group differences
Flemmen et al., 2014 [19]	Substance Use Disorders	Hospital Anxiety and Depression Scale (HAD)	Placebo exercise	9/7	11/28.5	33/31	3	8	25	treadmill	240/240	90–95% of HRmax	~70% max HR	HIIT improved depressive symptoms. No between-group differences
Gerber et al., 2018 [12]	MDD	The German version of the Beck Depression Inventory II (BDI-II)	MIT	25/25	76/80	36.4/36.5	3	4	30	bike	30/30	80% of VO_2_max	passive rest	Both groups improved. No between-group difference
Plag et al., 2020 [18]	Anxiety disorders	Hamilton Rating Scale for Depression (HAM-D)	Low Intensity exercise	17/16	72.7/68.7	40.18/41.94	every second day	12 days	20	bike	60/60	77–95% max HR	<70% max HR	Both groups improved. Stronger reduction in HIIT compared to LIT

MDD: Major depressive disorder; MIT: moderate intensity training; HIIT: high-intensity interval training; peak HR: peak heart rate; HRmax: maximum heart rate; VO_2_max: maximum oxygen consumption.

## Data Availability

Not Applicable.

## Supplementary Materials

The following supporting information can be downloaded at: https://www.mdpi.com/article/10.3390/ijerph191710581/s1. Figure S1. PRISMA Flowchart of study selection.
