# Supplementary figures and images for: A Rapid Review of Randomized Trials Assessing the Effects of High-Intensity Interval Training on Depressive Symptoms in People with Mental Illness
Source: Int J Environ Res Public Health. 2022 Aug 25;19(17):10581. doi: 10.3390/ijerph191710581 (PMC9518083; doi:10.3390/ijerph191710581)

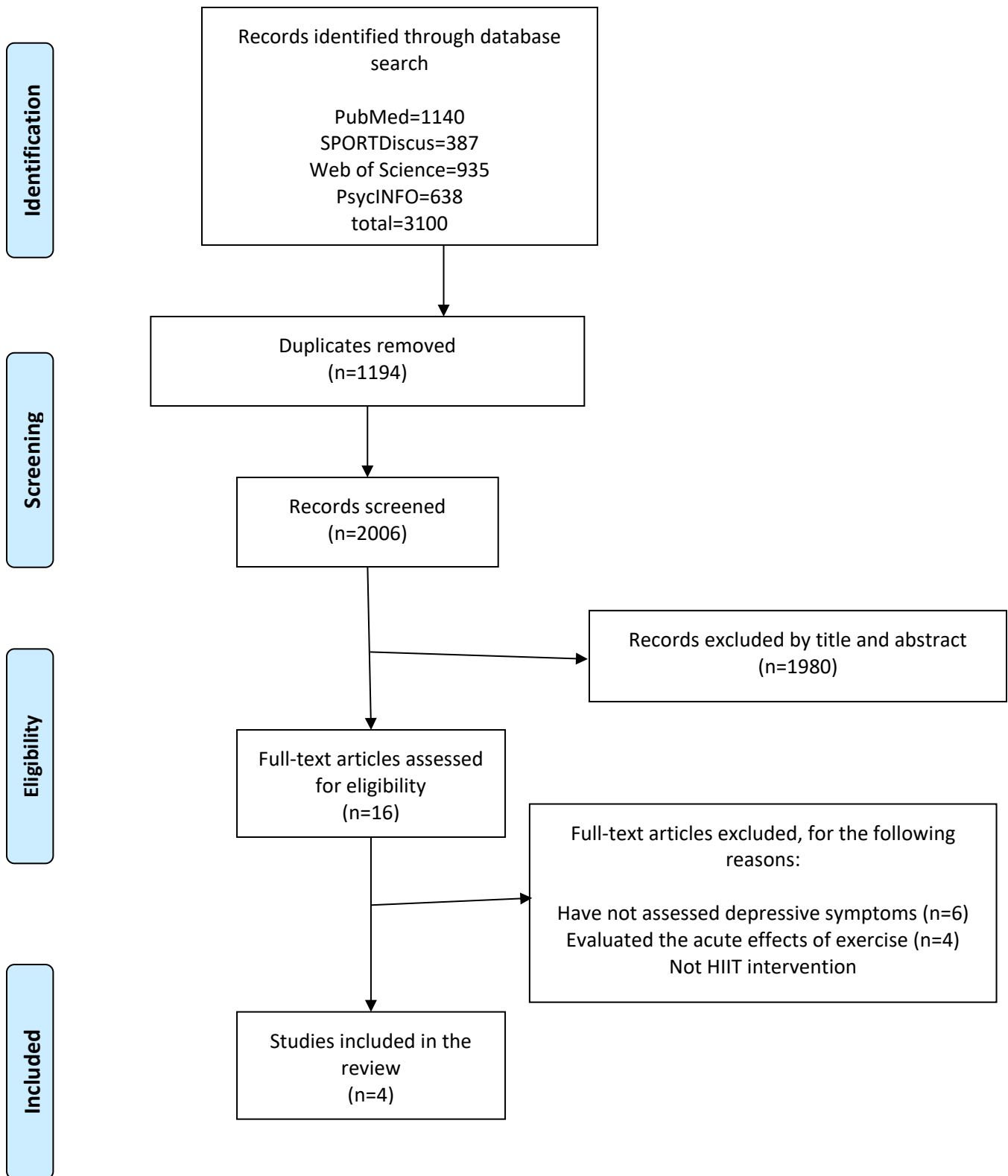

Figure S1. PRISMA Flowchart of study selection

Supplement: Supplementary file 1 [file ijerph-19-10581-s001.zip › ijerph-1844665-supplementary.pdf]
